# Population structure and genetic diversity of *Toona sinensis* revealed by whole-genome resequencing

**DOI:** 10.1186/s12863-024-01288-w

**Published:** 2025-01-03

**Authors:** Lei Wang, Chang Lu, Zhi-Gang Bao, Meng Li, Fusheng Wu, Yi-Zeng Lu, Bo-Qiang Tong, Mei Yu, Yong-Jun Zhao

**Affiliations:** 1 Key Laboratory of State Forestry and Grassland Administration Conservation and Utilization of Warm Temperate Zone Forest and Grass Germplasm Resources, Shandong Provincial Center of Forest and Grass Germplasm Resources, Ji’nan, 250103 Shandong China; 2 College of Food Science and Engineering, Shandong Agriculture and Engineering University, Ji’nan, 250103 China

**Keywords:** *Toona sinensis*, Whole-genome resequencing, Genome assembly, Genetic diversity, Population structure

## Abstract

**Objectives:**

*Toona sinensis*, commonly known as Chinese toon, is a perennial woody plant with significant economic and ecological importance. This study employed whole-genome resequencing of 180 T*. sinensis* samples collected from Shandong to analyze genetic variation and diversity, ultimately identifying 18,231 high-quality SNPs after rigorous quality control and linkage disequilibrium pruning. This comprehensive genomic resource provides novel insights into the genetic architecture of *T. sinensis*, facilitating the elucidation of population structure and supporting future breeding programs.

**Data description:**

We performed whole-genome resequencing on 180 *Toona sinensis* samples, generating 1170.26 Gbp of clean data with a Q30 percentage of 93.69%. The average alignment rate to the reference genome was 96.72%, with an average coverage depth of 8 × and a genome coverage of 88.71%. Following data quality control and alignment, we performed SNP calling and filtering to identify high-quality SNPs across all samples. Population structure analyses were then conducted using the identified SNPs, including principal component analysis (PCA), structure analysis, and phylogenetic tree construction. These comprehensive analyses provide a foundation for understanding the genetic diversity and evolutionary dynamics of *T. sinensis*.

## Objective

*Toona sinensis,* a member of the Meliaceae family, is widely cultivated for its edible young leaves, medicinal properties, strong adaptability, and resilience [1]. This plant is significant for both ecological restoration and agricultural applications. The young leaves of *T. sinensis* are not only highly nutritious but have also been traditionally recognized for their health benefits, including anti-inflammatory and anti-cancer properties [2].

Despite its significant economic and medicinal value, the genetic diversity and population structure of *T. sinensis* have yet to be thoroughly elucidated, hindering efficient conservation and breeding programs. Population structure analyses, including principal component analysis (PCA), ADMIXTURE, and phylogenetic tree construction, indicated the presence of four distinct genetic subgroups among the samples. These findings highlight the genetic diversity within *T. sinensis* populations, providing valuable insights for future breeding and conservation of this economically important species.

## Data description

We sampled 180 *Toona sinensis* individuals (Table 1, Data file 1) [3] from Shandong Province, China, to assess intraspecific genetic diversity. Resequencing these samples using an Illumina platform generated a total of 1170.26 Gb of clean data (Table 1, Data file 2) [4], with a Q30 score of 93.69%. Clean reads were then aligned to the *T. sinensis* reference genome (Table 1, Data file 3) [5] using the Burrows-Wheeler Alignment (BWA) [6] tool. The average alignment rate was 96.72%, with an average coverage depth of 8 × and a genome coverage of 88.71%.

**Table 1:** Overview of data files/data sets

Label	Name of data file/data set	File types	Data repository and identifier
Data file 1	Sample information	Dataset (.xlsx)	li, leilei. Figshare. (2024) https://doi.org/10.6084/m9.figshare.27224772 [3]
Data file 2	Illumina resequencing reads	Fastq file (fq.gz)	China National GeneBank DataBase (Accession no: CNP0006008) [4]
Data file 3	Reference genome assemble	Fasta file (fasta.gz)	China National GeneBank DataBase (Accession no: CNA0019196) [7]
Data file 4	Genotyping data before filtering	VCF file (vcf.gz)	li, leilei. Figshare. (2024) https://doi.org/10.6084/m9.figshare.27224772.v3. [3]
Data file 5	SNP data comprising 18,231 variants	VCF file (vcf.gz)	li, leilei. Figshare. (2024) https://doi.org/10.6084/m9.figshare.27224772.v3. [3]
Data file 6	Figure 1	PDF file (.pdf)	li, leilei. Figshare. (2024) https://doi.org/10.6084/m9.figshare.27224772.v3. [3]

We employed the Genome Analysis Toolkit (GATK) [8] for variant calling to detect single nucleotide polymorphisms (SNPs) (Table 1, Data file 4) [3] and filtered them using the following parameters: QD < 2.0, MQ < 40.0, FS > 60.0, QUAL < 30.0 and MQrankSum < -12.5. After performing linkage disequilibrium pruning using vcftools (v.0.1.15) [9], a total of 18,231 high-confidence SNPs (Table 1, Data file 5) [3] were obtained for further population structural analyses. To explore the genetic structure of *T. sinensis* populations, we employed the ADMIXTURE software [10], utilizing the 18,231 high-confidence SNPs. Testing was conducted on ancestral population numbers (K) ranging from 1 to 10 to infer the optimal population structure.

**Fig. 1:**
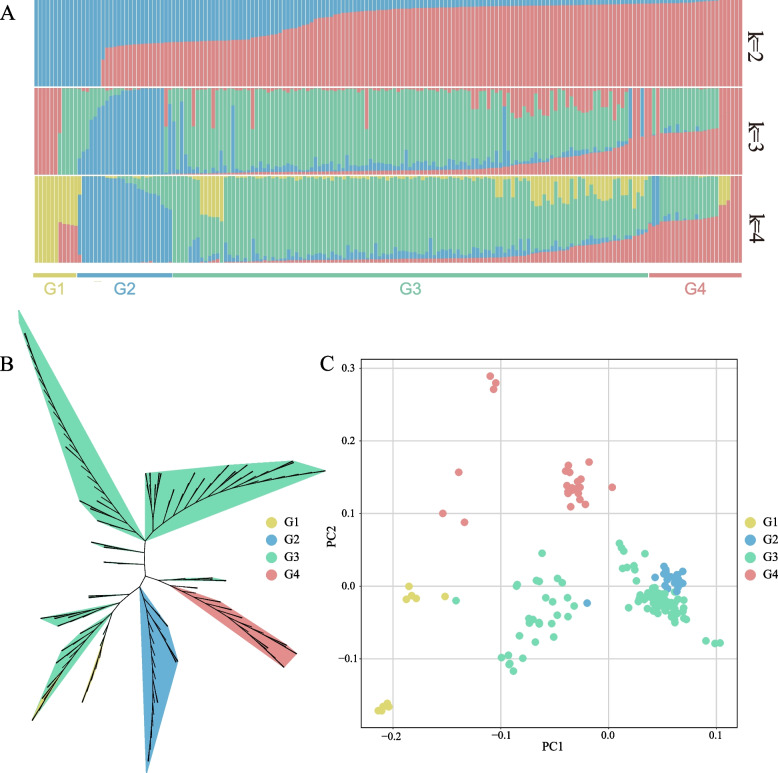
Population structure of 180 accessions of *Toona sinensis*. **A** Plots of *T. sinensis* individual ancestry inference for K = 2 to 4 based on 18,231 SNPs. **B** PCA plots of the first two components of 108 accessions. **C** Phylogenetic tree of all accessions inferred from 18,231 SNPs

The analysis indicated an optimal population structure at *K* = 4, resulting in the identification of four unique genetic subpopulations, denoted as G1-G4 **(**Fig. 1A**)**. G3 is the largest group, exhibiting a significant number of individuals with admixed ancestry, specifically with G4. G2 demonstrated a relatively consistent genetic composition with limited admixture. G1, having the smallest population size, comprised some individuals with unique genetics while others exhibited admixture with G3.

A neighbor-joining phylogenetic tree was constructed with 1,000 bootstrap replicates using MEGA X software [11] to investigate phylogenetic relationships among *T. sinensis* accessions based on the 18,231 SNP dataset, and it was further visualized using the ggtree package [12] in R. The resulting tree validated the population structure findings, as the accessions clustered into four distinct groups corresponding to G1-G4 (Fig. 1B). The G3 lineage occupied the largest area of the phylogenetic tree, indicating its extensive genetic diversity.

The findings of the population structure were further substantiated by PCA, as presented in Fig. 1C. A clear demarcation into four key clusters was evidenced in the PCA plot, corroborating the results obtained from the ADMIXTURE and phylogenetic analyses. Finally, the comprehensive results obtained from this study suggest that the *T. sinensis* population can be classified into four predominant genetic lineages. The G3 population exhibits extensive genetic diversity and admixture levels, likely reflecting heightened gene flow. The G2 lineage demonstrates a relatively homogeneous genetic structure, while the G1 lineage, characterized by the smallest effective population size, exhibits unique genetic characteristics in certain individuals and introgression with other lineages in some individuals. These findings provide valuable insights into the evolutionary history, genetic diversity and evolution of *T. sinensis* populations.

## Limitations

The primary limitation of this study is the average coverage depth of 8X, which may not be sufficient for detecting rare variants with high confidence. Additionally, the study focuses on a specific set of samples, which may not represent the full genetic diversity of *Toona sinensis* across its entire geographical range. Future studies with higher coverage and a broader sample collection are necessary to gain a more comprehensive understanding of the genetic diversity of this species.

## Data Availability

The raw sequencing data are available at the China National GeneBank DataBase (CNGBdb) with the accession number CNP0006008. Data files 4 and 5 listed Table 1 are freely and openly accessible on Figshare (10.6084/m9.figshare.27224772.v3 ).

## Abbreviations

PCA: Principal component analysis
SNP: Single Nucleotide Polymorphism
GATK: Genome Analysis Toolkit
BWA: Burrows-Wheeler Alignment
